# Antioxidative Characteristics of *Anisomeles indica* Extract and Inhibitory Effect of Ovatodiolide on Melanogenesis

**DOI:** 10.3390/ijms13056220

**Published:** 2012-05-21

**Authors:** Huey-Chun Huang, Hsiu-Man Lien, Hui-Ju Ke, Li-Ling Chang, Chia-Chang Chen, Tsong-Min Chang

**Affiliations:** 1 Department of Medical Laboratory Science and Biotechnology, China Medical University, No, 91 Hsueh-Shih Road, Taichung 40402, Taiwan; E-Mail: lchuang@mail.cmu.edu.tw; 2 Department of Chemistry, Tung Hai University, No, 181, Section 3, Taichung Port Road, Taichung 40704, Taiwan; E-Mail: herb736@yahoo.com.tw; 3 Department of Applied Cosmetology & Master Program of Cosmetic Science, Hung Kuang University, No. 34, Chung-Chie Rd., Shalu, Taichung 43302, Taiwan; E-Mails: huiju330@gmail.com (H.-J.K.); liling0706@gmail.com (L.-L.C.); 4 School of Management Department, Feng Chia University, No, 100, Wenhwa Road, Seatwen, Taichung 40724, Taiwan; E-Mail: yspharm@ms14.hinet.net

**Keywords:** *Anisomeles indica*, ovatodiolide, melanogenesis, tyrosinase, melanin, antioxidant

## Abstract

The purpose of the study was to investigate the antioxidant characteristics of *Anisomeles indica* methanol extract and the inhibitory effect of ovatodiolide on melanogenesis. In the study, the antioxidant capacities of *A. indica* methanol extract such as DPPH assay, ABTS radical scavenging assay, reducing capacity and metal ion chelating capacity as well as total phenolic content of the extract were investigated. In addition, the inhibitory effects of ovatodiolide on mushroom tyrosinase, B16F10 intracellular tyrosinase and melanin content were determined spectrophotometrically. Our results revealed that the antioxidant capacities of *A. indica* methanol extract increased in a dose-dependent pattern. The purified ovatodiolide inhibited mushroom tyrosinase activity (IC_50_ = 0.253 mM), the compound also effectively suppressed intracellular tyrosinase activity (IC_50_ = 0.469 mM) and decreased the amount of melanin (IC_50_ = 0.435 mM) in a dose-dependent manner in B16F10 cells. Our results concluded that *A. indica* methanol extract displays antioxidant capacities and ovatodiolide purified from the extract inhibited melanogenesis in B16F10 cells. Hence, *A. indica* methanol extract and ovatodiolide could be applied as a type of dermatological whitening agent in skin care products.

## 1. Introduction

Free radicals are atoms or molecules which carry an unpaired electron. For example, the superoxide anion, hydroxyl radical and peroxyl radical are three common short-lived and chemically reactive free radicals. Additionally, reactive oxygen species (ROS) such as singlet oxygen, molecular oxygen and hydrogen peroxide (H_2_O_2_) are not free radicals, but are able to initiate oxidative reactions and generate free-radical species. Certainly, the cellular enzymes and controlled metabolic pathways ordinarily keep cellular oxidative damage to a minimum level [1–3]. Furthermore, it is reported that UV light radiation causes protein oxidation, DNA damage and induces the synthesis of ROS in skin, which in turn induces aging-related disorders or melanogenesis. Therefore, the use of natural botanical antioxidants to protect human skin from the harmful effects of UV radiation is highly topical, having attracted increasing interest in recent years [4].

Melanin is a pigment produced by melanocytes and is responsible for skin color as well as protecting the skin from UV-induced injury [5]. However, overproduction or accumulation of melanin results in several dermatological disorders, including melasma, age spots, freckles, and other hyperpigmentation syndromes [6]. In the processes of melanin biosynthesis, tyrosinase is the key enzyme in the rate-limiting step in which l-tyrosine is hydroxylated to l-DOPA (*O*-diphenol product), and l-DOPA is further oxidized into the corresponding *O*-quinone [7]. Hence, tyrosinase is a major target in screening inhibitors of melanin synthesis. Besides, it is reported that l-DOPA could also autoregulate itself and regulate the melanocyte functions through intermediates of melanogenesis and through the activity of structural or regulatory melanocytes [8]. Furthermore, it has been reported that microphthalmia-associated transcription factor (MITF) and other enzymes such as tyrosinase related protein-1 (TRP-1) and tyrosinase related protein-2 (TRP-2) also contribute to the production of melanin [9–11]. Recently, many melanogenesis inhibitors have been growingly applied in skin care cosmetics for the prevention of hyperpigmentation [12]. Interestingly, melanogenesis is reported to produce hydrogen peroxide and other ROS which subjects the melanocytes under high-grade oxidative stress. It is reported that ROS play a significant role in the regulation of melanogenesis, while ROS scavengers and inhibitors of ROS generation may down-regulate UV-induced melanogenesis [13]. Therefore, several antioxidants, such as ascorbic derivatives and reduced glutathione (GSH), have been applied as inhibitory agents of melanogenesis [14,15].

*Anisomeles indica* Kuntze (Labiatae) is a specific woody shrub in Taiwan and has been commonly used in traditional Chinese medicines for various disorders such as gastrointestinal disorders, liver disease and inflammatory skin diseases [16]. It is also reported that *A. indica* extracts and isolated constituents inhibit inflammatory mediators and tumor cell proliferation [17–19]. Furthermore, the aqueous extract of *A. indica* has been shown to show anti-histaminerigic, anti-hyperalgesic and analgesic activities [20,21]. Recently, the ethanol extract of *A. indica* exhibited anti-bacterial activity [22]. However, there is no scientific report about the dermatological application of the extract or pure compound of this plant. The aim of this study was to investigate the antioxidative characteristics of *A. indica* methanol extract and evaluate the inhibitory effect of ovatodiolide on melanogenesis in B16F10 melanoma cells.

## 2. Results and Discussion

### 2.1. Purification of Ovatodiolide from *A. indica* Methanol Extract

**Figure 1:**
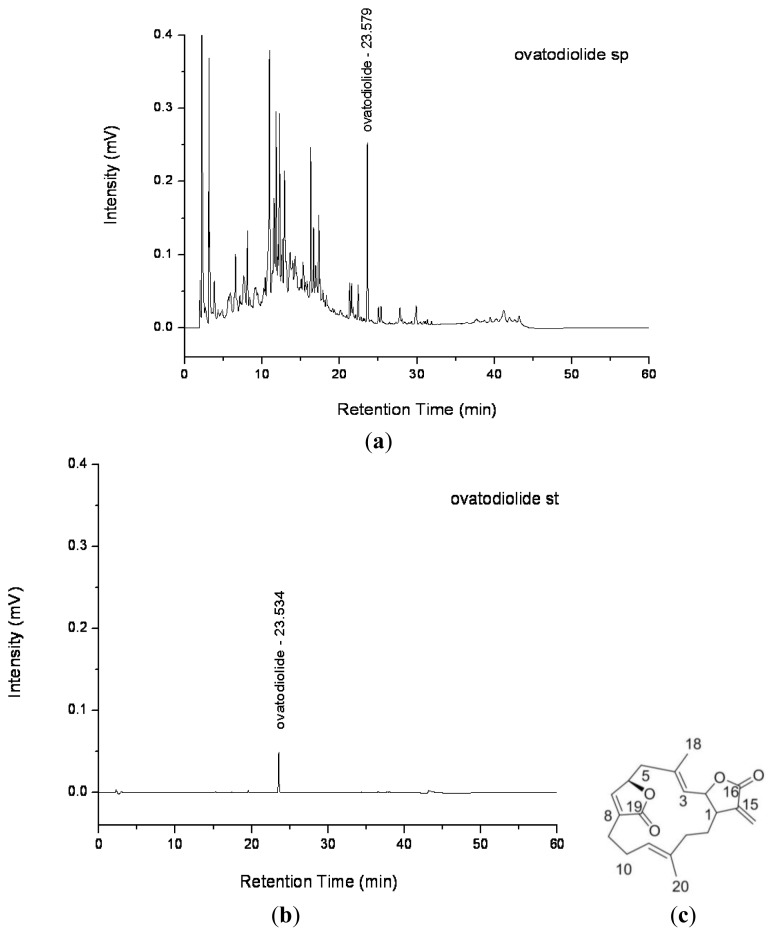
(**a**), (**b**) HPLC chromatogram and (**c**) chemical structure of ovatodiolide.

The HPLC chromatogram of *A. indica* methanol extract and ovatodiolide standard are shown in Figure 1. The retention time for purified ovatodiolide and standard were 23.579 and 23.534 min, respectively, which indicated the chemical purified from *A. indica* methanol extract was ovatodiolide. The chemical structure of ovatodiolide is shown in Figure 1c.

### 2.2. Determination of DPPH Scavenging Activity

**Figure 2:**
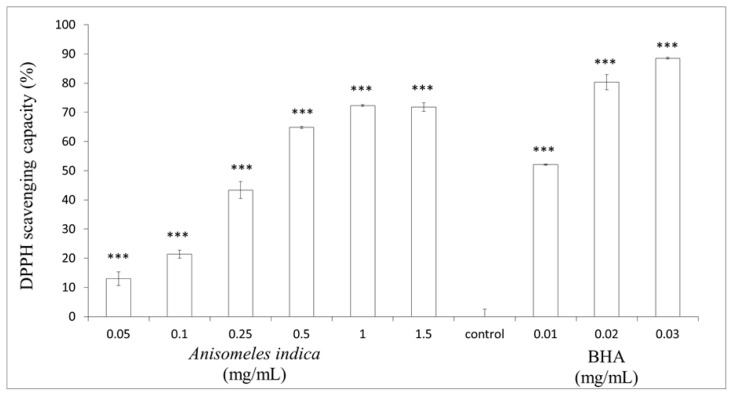
DPPH radical scavenging effects of *Anisomeles indica* methanol extract. Various concentrations of the extract (final concentration 0.05, 0.1, 0.25, 0.5, 1.0 and 1.5 mg/mL) or BHA (0.01, 0.02 and 0.03 mg/mL) were interacted with DPPH, respectively. Results are represented as percentages of control, and the data are mean ± SD for three separate experiments. Values are significantly different by comparison with control. ^***^ *p* < 0.001.

The antioxidant activity of *A. indica* methanol extract was first determined by measuring the DPPH scavenging ability of the extract. The extract showed DPPH radical scavenging activity as shown in Figure 2. DPPH scavenging activity of 0.05, 0.1, 0.25, 0.5, 1.0 and 1.5 mg/mL of the extract was 12.94 ± 2.31%, 21.42 ± 1.36%, 43.34 ± 2.94%, 64.86 ± 0.35%, 72.31 ± 0.34% and 71.79 ± 1.49% (*p* < 0.001) of control, respectively. Meanwhile, the DPPH scavenging activity of 0.01, 0.02 and 0.03 mg/mL of BHA was 52.04 ± 0.19%, 80.34 ± 2.61% and 88.54 ± 0.36% (*p* < 0.001) of control, respectively. Our results indicate that 0.05–1.0 mg/mL of *A. indica* methanol extract display DPPH free radical scavenging activity in a dose-dependent pattern. DPPH assay is a known useful method to give reliable information with regard to screening the antioxidant capacity of specific compounds or plant extracts. The results shown in Figure 2 imply that *A. indica* methanol extract displays DPPH free radical scavenging activity in a dose-dependent manner. The steric accessibility of the DPPH radical is a major determinant of the assay reaction, since some small molecules that have better access to the radical site could show higher antioxidant capacity. Besides, many large antioxidant chemicals that react quickly with peroxyl radicals may react slowly in a DPPH assay [23]. Additionally, the spectrophotometric analysis can be affected by the chemical structure of test compounds that absorb at the wavelength of determination or by the turbidity of the sample.

### 2.3. Determination of ABTS^+^ Radical Scavenging Capacity

**Figure 3:**
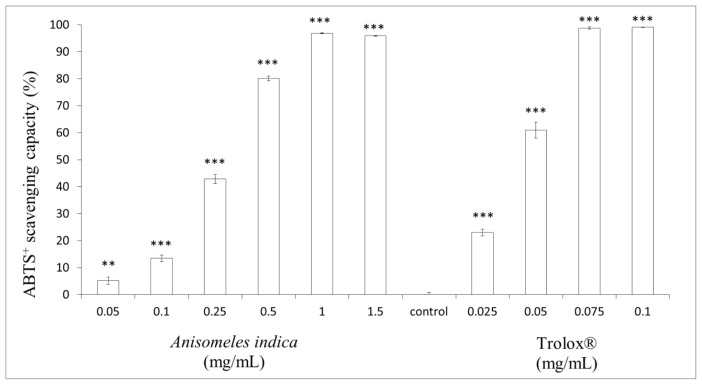
ABTS^+^ radical scavenging effects of *A. indica* methanol extract. The extract (final concentration 0.05, 0.1, 0.25, 0.5, 1.0 and 1.5 mg/mL) or Trolox^®^ (0.025, 0.05, 0.075 and 0.1 mg/mL) were interacted with ABTS. Results are represented as percentages of control, and the data are mean ± SD for three separate experiments. Values are significantly different by comparison with control. ^***^ *p* < 0.001.

The ABTS^+^ assay was employed to confirm the antioxidant activity of *A. indica* methanol extract. Different concentrations of the methanol extract (final concentration 0.05, 0.1, 0.25, 0.5, 1.0 and 1.5 mg/mL) or Trolox^®^ (0.025, 0.05, 0.075 and 0.1 mg/mL) were incubated with ABTS^+^ solution, respectively. The ABTS^+^ scavenging capacity of the methanol extract was 5.14 ± 1.36%, 13.42 ± 1.27%, 42.84 ± 1.63%, 80.13 ± 0.91%, 96.83 ± 0.14% and 95.86 ± 0.09% of control for the extract at the concentration of 0.05, 0.1, 0.25, 0.5, 1.0 and 1.5 mg/mL, respectively (*p* < 0.001). Meanwhile, the ABTS^+^ scavenging capacity of Trolox^®^ (0.025, 0.05, 0.075 and 0.1 mg/mL) was 23.04 ± 1.22%, 61.00 ± 2.96%, 98.78 ± 0.40% and 99.05 ± 0.03%, respectively (*p* < 0.001). The results indicated that the methanol extract of *A. indica* scavenges ABTS^+^ free radical significantly in a dose-dependent manner (0.05–1.0 mg/mL). Additionally, the maximum ABTS^+^ radical scavenging capacity of methanol extract was slight weaker than that of Trolox^®^ (Figure 3). The ABTS^+^ spectrophotometric analysis is a technically easy and simple method to screen potential antioxidant. The results shown in Figure 3 indicate that the methanol extract of *A. indica* scavenges ABTS^+^ free radical significantly in a dose-dependent pattern (0.05–1.0 mg/mL). Thermodynamically, any sample that has a redox potential lower than that of ABTS^+^ may react with the radical. Furthermore, the ABTS^+^ radical is soluble in water and organic solvents, accounting for the determination of antioxidant capacity of hydrophilic and lipophilic samples. Furthermore, the results provided by the assay are dependent on time of analysis.

### 2.4. Measurement of Reducing Capacity

**Figure 4:**
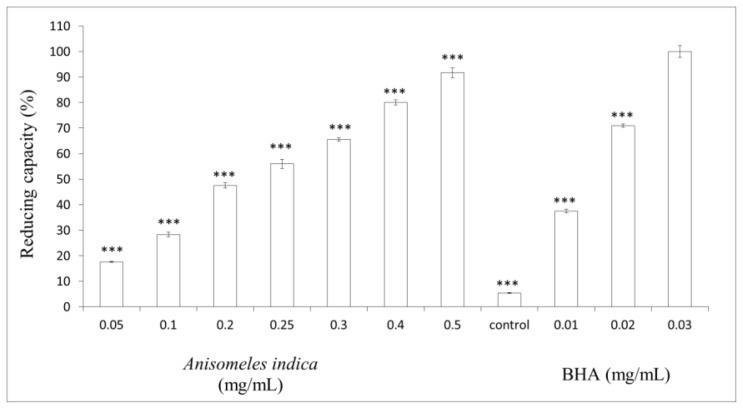
Reducing capacity of *A. indica* methanol extract. Different concentrations of the extract (0.05, 0.1, 0.2, 0.25, 0.3, 0.4 and 0.5 mg/mL) or BHA (0.01, 0.02, 0.03 mg/mL) were used in the assay. Results are represented as percentages of control, and the data are mean ± SD for three separate experiments. Values are significantly different by comparison with control. ^***^ *p* < 0.001.

To determine the reducing capacity of *A. indica* methanol extract, various concentrations of the extract (0.05, 0.1, 0.2, 0.25, 0.3, 0.4 and 0.5 mg/mL) or BHA (0.01, 0.02, 0.03 mg/mL) were tested. The results shown in Figure 3 reveal that *A. indica* methanol extract exhibit reducing capacity in a dose-dependent pattern. Higher concentrations of *A. indica* methanol extract present apparent reducing capacity. The reducing capacities of 0.3, 0.4 and 0.5 (mg/mL) of the methanol extract were 65.53 ± 0.63%, 80.07 ± 1.05% and 91.7 ± 1.91%, respectively. Meanwhile, the reducing capacities of 0.01, 0.02 and 0.03 (mg/mL) of BHA were 37.48 ± 0.72, 70.98 ± 0.68, 37.48 ± 0.72 and 100 ± 2.35%, respectively (Figure 4). In this assay, the yellow color of the test solution changes to various shades of green and blue, depending on the reducing capacity of the test sample.

### 2.5. Measurement of Metal Ion-Chelating Capacity

**Figure 5:**
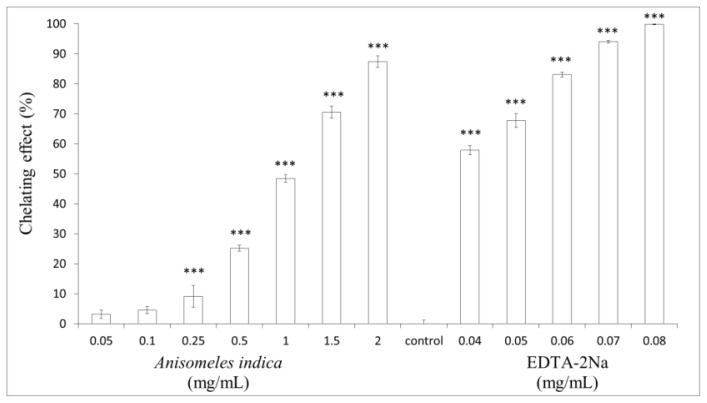
Metal-ion chelating activity of *A. indica* methanol extract. Different concentrations of the extract (0.05, 0.1, 0.25, 0.5, 1.0, 1.5 and 2.0 mg/mL) or EDTA (0.04–0.08 mg/mL) were used in the study. Results are represented as percentages of control, and the data are mean ± SD for three separate experiments. Values are significantly different by comparison with control. ^***^ *p* < 0.001.

Antioxidants may form insoluble metal complexes with ferrous ions and then inhibit interaction between metal and lipid. The metal-ion chelating ability of *A. indica* methanol extracts (0.05, 0.1, 0.25, 0.5, 1.0, 1.5 and 2.0 mg/mL) were 3.17 ± 1.37%, 4.54 ± 1.15%, 9.13 ± 3.64%, 25.18 ± 0.99%, 48.48 ± 1.28%, 70.6 ± 1.98% and 87.41 ± 1.89% of control, respectively (*p* < 0.001). On the other hand, the metal-ion chelating capacity of 0.04, 0.05, 0.06, 0.07 and 0.08 mg/mL of EDTA were 57.88 ± 1.55%, 67.72 ± 2.35%, 83.04 ± 0.81%, 94.06 ± 0.43% and 99.85 ± 0.11%, respectively (*p* < 0.001) (Figure 5). The results shown in Figure 5 indicate the dose-dependent metal-ion chelating capacity of *A. indica* methanol extract, and the extract may contain some special components that can chelate metal ions. The presence of antioxidants in the extract may cause the reduction of the Fe^3+^ ferricyanide complex to the ferrous form. Hence, measuring the formation of blue color at 700 nm can monitor the Fe^2+^ concentration [24]. The results shown in Figure 5 reveal that *A. indica* methanol extract chelate metal-ions in a dose-dependent pattern. Hence, the extract may contain some special components that can chelate metal ions.

### 2.6. Measurement of Total Phenol Content

**Figure 6:**
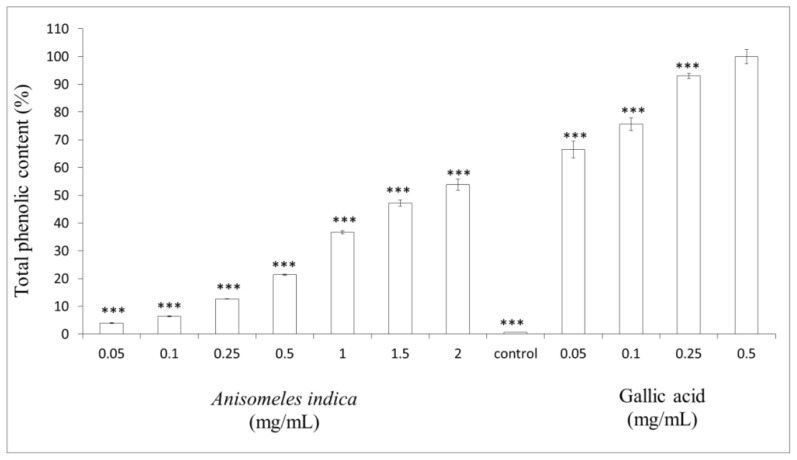
Determination of total phenolic content. Different concentrations of *A. indica* methanol extract (0.05, 0.1, 0.25, 0.5, 1.0, 1.5 and 2.0 mg/mL) and gallic acid (0.05, 0.1, 0.25, 0.5 mg/mL) were used in the assay. Results are represented as percentages of control, and the data are mean ± SD for three separate experiments. Values are significantly different by comparison with control. ^***^ *p* < 0.001.

To determine the amount of total phenolic contents of *A. indica* methanol extract (0.05, 0.1, 0.25, 0.5, 1.0, 1.5 and 2.0 mg/mL), gallic acid (0.05, 0.1, 0.25 and 0.5 mg/mL) was used as positive standard. The results in Figure 6 show that the total phenolic contents in *A. indica* methanol extract were less than those of gallic acid. The phenolic content of 2.0 mg/mL of the methanol extract (53.90 ± 2.02%) was still less than that of 0.05 mg/mL of gallic acid (66.53 ± 3.02%).

### 2.7. Inhibitory Effect of *A. indica* Methanol Extract and Ovatodiolide on Mushroom Tyrosinase Activity

**Figure 7:**
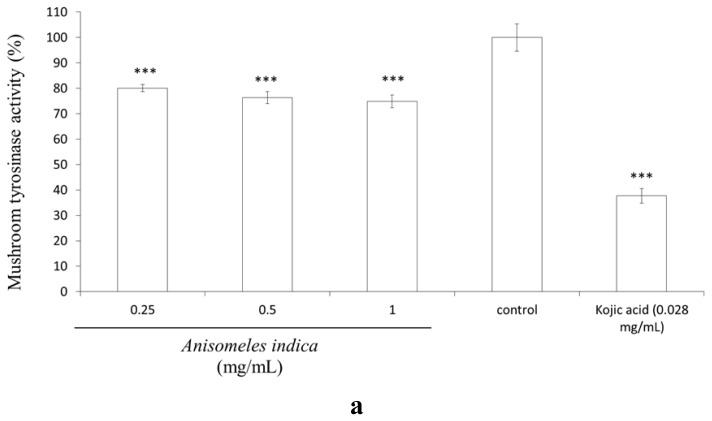

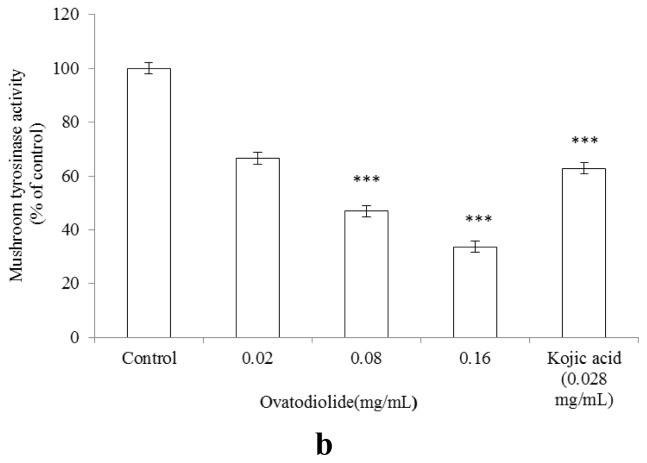
Inhibitory effect of *A. indica* methanol extract and ovatodiolide on mushroom tyrosinase activity. Different concentrations of (**a**) the methanol extract (0.25, 0.5, 1.0 mg/mL); (**b**) ovatodiolide (0.02, 0.08 and 0.16 mg/mL) or kojic acid (0.028 mg/mL) were incubated with the same units of mushroom tyrosinase. Results are represented as percentages of control, and data are presented as mean ± SD for three separate experiments. Values are significantly different by comparison with control. ^***^ *p* < 0.001.

In order to assay the possible inhibitory effect of *A. indica* methanol extract on mushroom tyrosinase activity, dose-dependent inhibition experiments were carried out in triplicate. The results shown in Figure 7a indicate that mushroom tyrosinase activity was slightly inhibited by the various concentrations of *A. indica* methanol extract (final concentration 0.25, 0.5 and 1.0 mg/mL) in a dose-dependent manner. The remained tyrosinase activity was 80.05 ± 1.38%, 76.36 ± 2.37% and 74.86 ± 2.49% of control for 0.25, 0.5 and 1.0 (mg/mL) of the methanol extract, respectively (*p* < 0.001). Meanwhile, mushroom tyrosinase activity was inhibited by kojic acid (0.028 mg/mL) and residual enzyme activity was 64.37 ± 3.50% of control (*p* < 0.001). Hence, *A. indica* methanol extract seems to act as a tyrosinase inhibitor. Interestingly, the results shown in Figure 7b indicate ovatodiolide displays a more potent inhibitory effect on mushroom tyrosinase activity than kojic acid (0.028 mg/mL). The observed mushroom tyrosinase activity was 66.60 ± 2.13%, 46.97 ± 2.21% and 33.69 ± 1.08% of control for 0.02, 0.08 and 0.16 (mg/mL) of ovatodiolide, respectively (*p* < 0.001). The IC_50_ of ovatodiolide on mushroom tyrosinase activity is equal to 0.253 mM. The results shown in Figure 7a indicate that mushroom tyrosinase activity was slightly inhibited by the various concentrations of *A. indica* methanol extract (final concentration 0.25, 0.5 and 1.0 mg/mL) in a dose-dependent manner. Actually, we also determined the effect of *A. indica* methanol extract on intracellular tyrosinase activity and melanin content in B16F10 cells. However, the extract displayed minor inhibitory effects on melanogenesis in B16F10 melanoma. In addition, the water extract of *A. indica* neither inhibits mushroom tyrosinase nor suppresses intracellular tyrosinase activity in B16F10 cells (data not shown).

### 2.8. Inhibitory Effect of Ovatodiolide on Melanin Production

**Figure 8:**
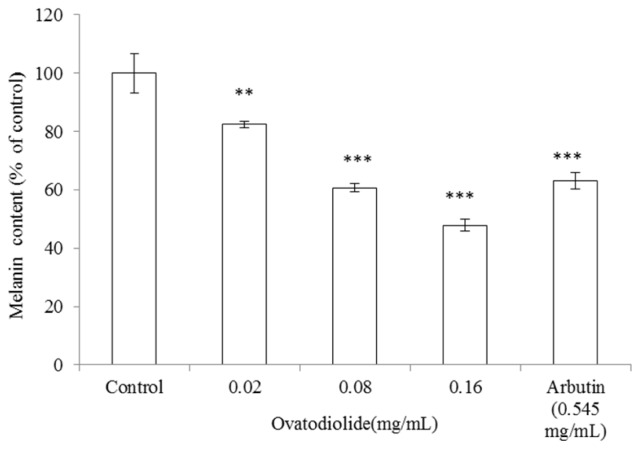
Effect of ovatodiolide on melanin synthesis in B16F10 cells. Melanin content assessment was performed as described in the “Experimental Section”. Briefly, cells were cultured with α-MSH (100 nM) for 24 h, and then the melanin content was measured after treatment with various concentrations of ovatodiolide (0.02, 0.08 and 0.16 mg/mL) or arbutin (0.545 mg/mL) for another 24 h. Results are represented as percentages of control, and data are presented as mean ± SD for three separate experiments. Values are significantly different by comparison with control. ^***^ *p* < 0.001.

To determine the antimelanogenic activity of ovatodiolide, the inhibitory effect of ovatodiolide on melanin content in B16F10 melanoma cells was assayed. B16F10 cells were first stimulated with α-MSH (100 nM) for 24 h, and then cultured in the presence of ovatodiolide at 0.02, 0.08 and 0.16 (mg/mL) or arbutin (0.545 mg/mL), respectively. Treatment with ovatodiolide showed a significant inhibitory effect on melanin synthesis in a dose-dependent pattern. The melanin content was represented as percentage of control. After treatment, the melanin content was 82.39 ± 1.18%, 60.66 ± 1.47% and 47.83 ± 2.03% for 0.02, 0.08 and 0.16 (mg/mL) of ovatodiolide, respectively (*p* < 0.001) (Figure 8). The IC_50_ of ovatodiolide on B16F10 melanin content is 0.435 mM. Meanwhile, B16F10 cells were treated with arbutin (0.545 mg/mL) as positive standard, and the remaining intracellular melanin content was 63.03 ± 2.91% of control for arbutin (*p* < 0.001).

### 2.9. Inhibitory Effect of Ovatodiolide on B16F10 Tyrosinase Activity

**Figure 9:**
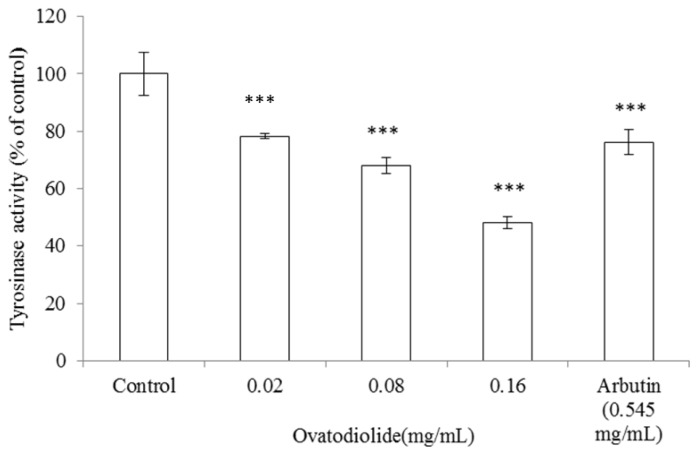
Effect of ovatodiolide on tyrosinase activity in B16F10 cells. Enzyme assay was performed as described in the “Experimental Section”. Briefly, B16F10 melanoma cells were stimulated with α-MSH (100 nM) for 24 h, and the cellular tyrosinase activity was assayed after treatment with ovatodiolide (0.02, 0.08 and 0.16 mg/mL) or arbutin (0.545 mg/mL) for another 24 h. Results are represented as percentages of control, and the data are mean ± SD for three separate experiments. Values are significantly different by comparison with control. ^***^ *p* < 0.001.

To examine the action mechanism of the inhibitory effect of ovatodiolide on melanogenesis more precisely, we assessed intracellular tyrosinase activity in B16F10 melanoma cells. The cells were first stimulated with α-MSH (100 nM) for 24 h, and then cultured with various concentrations of ovatodiolide (0.02, 0.08 and 0.16 mg/mL) or arbutin (0.545 mg/mL) for another 24 h. Ovatodiolide significantly inhibited α-MSH-induced tyrosinase activity in a dose-dependent pattern. After these treatments, the remaining intracellular tyrosinase activity was 78.27 ± 0.80%, 67.97 ± 2.78% and 48.03 ± 2.08% for 0.02, 0.08 and 0.16 mg/mL of ovatodiolide, respectively (*p* < 0.001). The IC_50_ of ovatodiolide on B16F10 intracellular tyrosinase is 0.469 mM. Meanwhile, the intracellular tyrosinase activity was 76.19 ± 4.47% after the cells were treated with arbutin (2.0 mM = 0.545 mg/mL) (*p* < 0.001) (Figure 9). Recently, ovatodiolide has been reported to show several pharmacological activities, including HIV-inhibitory effects [25], antiplatelet aggregation activities [17], anti-inflammatory activities [19], anti-metastatic effects on human breast cancer cells [26] and induces apoptosis in human oral squamous cell carcinoma [27]. However, there are no reports about the dermatological application of ovatodiolide. This is the first report that ovatodiolide inhibits intracellular tyrosinase activity and decreases melanin content in a dose-dependent manner in the B16F10 melanoma cell model. In our unshown data, it was found that 0.008 mg/mL of ovatodiolide reduces 50% of intracellular ROS content in B16F10 cells. Hence, it could be proposed that ovatodiolide inhibits melanogenesis in B16F10 cells mediated by the depletion of intracellular ROS content. Certainly, the possible action mechanism of the inhibitory effect of ovatodiolide on melanogenesis will be elucidated in the near future.

## 3. Experimental Section

### 3.1. Chemicals and Reagents

Gallic acid, l-ascorbic acid (AA), Folin-Ciocalteau’s phenol reagent, 1,1-diphenyl-2-picrylhydrazyl (DPPH), butylated hydroxyanisole (BHA) and all other chemicals and solvents were obtained from Sigma-Aldrich (St. Louis, MO, USA) or Merck.

### 3.2. Plant Material, Extraction and Isolation of Pure Compound

The whole plants of *A. indica* were collected in September 2010 from the farm of Yusheng Co., Ltd. at Taichung in Taiwan, and a botanically identified voucher specimen (AIY-09-28) was deposited in the Graduate Institute of Cosmetic Science, Hung Kuang University, Taiwan. Extraction and separation of *A. indica* followed the previous procedures with slight modifications [19,28]. The air-dried whole plants (7.8 kg) of *A. indica* were extracted with methanol (10 L × 4) under reflux. After complete extraction, the combined extracts were concentrated under reduced pressure to give a dark brown syrup (296 g, 4.1% w/w). The crude extract was then suspended in H_2_O, defatted with *n*-hexane, and then partitioned with chloroform successively. The concentrated chloroform layer (59 g) was chromatographed on a silica gel column by eluting with hexane/ethyl acetate (EtOAc) gradient, with increasing polarity, and yielded six fractions (F1–F6). Fraction F4 was subjected to silica gel column chromatography (CC) eluted with different solvents of increasing polarity (*n*-hexane/EtOAc). Fraction F4 (5.5 g) was purified on a silica gel column using hexane/EtOAc (8:2) to yield Fraction 4–6 (523 mg). Fraction 4–6 was further separated by HPLC (CH_3_CN–0.1% TFA in H_2_O, 64:36, UV detection at 265 nm), and simultaneously yielded pure ovatodiolide (41 mg) (Figure 1).

### 3.3. Cell Culture

B16F10 cells (ATCC CRL-6475, from the BCRC Cell Line Bank, BCRC60031) were cultured in DMEM with 10% fetal bovine serum (FBS; Gibco BRL, Gaithersburg, MD, USA) and penicillin/streptomycin (100 IU/50 μg/mL) in a humidified atmosphere containing 5% CO_2_ in air at 37 °C. All the experiments were performed in triplicate and were repeated 3 times to ensure reproducibility.

### 3.4. DPPH Scavenging Activity Assay

The antioxidant activity of *A. indica* methanol extract was first determined by measuring the DPPH scavenging ability [29] as modified by Sánchez-Moreno *et al*. [30]. The methanol extract at various concentrations (final concentration 0.05, 0.1, 0.25, 0.5, 1.0 and 1.5 mg/mL) was added to 2.9 mL of DPPH (60 μM) solution. When DPPH reacts with an antioxidant that can donate hydrogen, it takes a reduced form, and the resulting decrease in absorbance at 517 nm was recorded using a UV-Vis spectrophotometer (Jasco, V-630, Tokyo, Japan). In this study, BHA (0.01, 0.02 and 0.03 mg/mL) was used as antioxidant standard.

### 3.5. ABTS^+^ Scavenging Capacity Assay

The ABTS decolonization assays were carried out as previously described [31] and involves the generation of ABTS^+^ chromophore by oxidation of ABTS with potassium persulfate. The ABTS radical cation (ABTS^+^) was produced by reacting 7 mM stock solution of ABTS with 2.45 mM potassium persulfate and allowing the mixture to stand in the dark for at least 6 h before use. Absorbance at 734 nm was measured 10 min after mixing different concentrations of *A. indica* methanol extract (final concentration 0.05, 0.1, 0.25, 0.5, 1.0 and 1.5 mg/mL) with 1 ml of ABTS^+^ solution. The ABTS^+^ scavenging capacity of *A. indica* methanol extract was compared with that of Trolox^®^ (0.025, 0.05, 0.075 and 0.1 mg/mL).

### 3.6. Determination of Reducing Capacity

The reducing power of *A. indica* methanol extract was determined according to the method of Oyaizu [32]. Different concentrations of *A. indica* methanol extract (0.05, 0.1, 0.2, 0.25, 0.3, 0.4 and 0.5 mg/mL) or BHA (0.01, 0.02, 0.03 mg/mL) were mixed with phosphate buffer (2.5 mL, 0.2 M, pH 6.6) and potassium ferricyanide [K_3_Fe(CN)_6_] (2.5 mL, 1% w/v). The mixture was incubated at 50 °C for 20 min. A portion (2.5 mL) of trichloroacetic acid (10% w/v) was added to the mixture, which was then centrifuged at 1000 g for 10 min. The upper layer of solution (2.5 mL) was mixed with distilled water (2.5 mL) and FeCl_3_ (0.5 mL, 0.1% w/v), and the absorbance was measured at 700 nm in a UV-Vis spectrophotometer. Higher absorbance of the reaction mixture indicated the greater reducing power of the test sample.

### 3.7. Measurement of Metal-Ion Chelating Capacity

The chelation of ferrous ions by the *A. indica* methanol extract or EDTA was determined by the previous method described by Dinis *et al*. [33] with slight modifications. Different concentrations of *A. indica* methanol extract (0.05–2.0 mg/mL) or EDTA (0.04, 0.05, 0.06, 0.07 and 0.08 mg/mL) was added to a solution of 1 mM FeCl_2_ (0.05 mL). Then 0.1 mL of ferrozine (1 mM) was added to the reaction mixture and the mixture was quantified to 1 mL with methanol, left standing at 25 °C for 10 min. The absorbance of the reaction mixture was measured at 562 nm. The percentage of chelating ability was calculated as follows: chelating ability (%) = (A_1_ − A_2_)/A_1_ × 100, where A_1_ is the absorbance of control and A_2_ is the absorbance in the presence of *A. indica* methanol extract or EDTA.

### 3.8. Determination of Total Phenolic Content

The amount of total phenolics of *A. indica* methanol extract was determined with the Folin–Ciocalteu reagent [34]. First, a standard curve was plotted using gallic acid as a standard (0.05, 0.1, 0.25, 0.5 mg/mL). Different concentrations of *A. indica* methanol extract (0.05, 0.1, 0.25, 0.5, 1.0, 1.5 and 2.0 mg/mL) were prepared in 80% of methanol. A 100 μL sample was dissolved in 500 μL (1/10 dilution) of the Folin-Ciocalteu reagent and 1000 μL of distilled water. The solutions were mixed and incubated at room temperature for 1 min. After 1 min, 1500 μL of 20% sodium carbonate solution was added. The final mixture was shaken and then incubated for 2 h in the dark at room temperature. The absorbance of samples was measured at 760 nm.

### 3.9. Assay of Mushroom Tyrosinase Activity

In order to assay the inhibitory action of *A. indica* methanol extract (0.25, 0.5 and 1.0 mg/mL) on mushroom tyrosinase activity, dose-dependent inhibition experiments were carried out in triplicate as described previously with a minor modification [35]. Briefly, 10 mL of aqueous solution of mushroom tyrosinase (200 units) were added to a 96-well microplate in a total volume of 200 μL mixture, containing 5 mM l-DOPA which is dissolved in 50 mM phosphate buffer (pH 6.8). The assay mixture was incubated at 37 °C for 30 min. Following incubation, the amount of dopachrome produced in the reaction mixture was determined spectrophotometrically at 490 nm (OD_490_) in a microplate reader. The inhibition percentage at three doses for each experiment was calculated by the following equation: inhibition percentage of tyrosinase activity (%) = (B − A) ÷ A × 100, where B is the mean of the measured OD_490_ values of the blank control, and A is the mean of the measured OD_490_ values for the *A. indica* methanol extract treated group.

### 3.10. Measurement of Melanin Content

The B16F10 intracellular melanin content was measured as described by Tsuboi *et al*. [36] with some modifications. The cells were treated with α-MSH (100 nM) for 24 h, and then the melanin content was determined after treatment with either ovatodiolide (final concentration 0.02, 0.08 and 0.16 mg/mL) or arbutin (0.545 mg/mL) for a further 24 h. After treatment, the cells were detached by incubation in trypsin/ethylenediaminetetraacetic acid (EDTA). After precipitation, cell pellets containing a known number of cells were solubilized in 1 N NaOH at 60 °C for 60 min. The melanin content was assayed by spectrophotometric analysis at an absorbance of 405 nm.

### 3.11. Assay of B16F10 Intracellular Tyrosinase Activity

Cellular tyrosinase activity was determined as described previously [37] with slight modifications. Briefly, the cells were treated with α-MSH (100 nM) for 24 h, and then intracellular tyrosinase activity was measured after treatment with various concentrations of ovatodiolide (final concentration 0.02, 0.08 and 0.16 mg/mL) or arbutin (0.545 mg/mL) for 24 h. After these treatments, the cells were washed twice with phosphate-buffered saline and homogenized with 50 mM PBS (pH 7.5) buffer containing 1.0% Triton X-100 and 0.1 mM PMSF. Intracellular tyrosinase activity was monitored as follows: Cell extracts (100 mL) were mixed with freshly prepared l-DOPA solution (0.1% in phosphate-buffered saline) and incubated at 37 °C. The absorbance at 490 nm was measured with microplate reader Gen 5™ (BIO-TEK Instrument, Bermont, USA) to monitor the production of dopachrome, corrected for auto-oxidation of l-DOPA.

### 3.12. Statistical Analysis

Statistical analysis of the experimental data points was performed by the one-way ANOVA test, which was used for comparison of measured data using SPSS 12.0 statistical software (4th Edition, SPSS INC. Chicago, USA, 2007). The data are presented as mean ± standard deviation of triplicate experiments. Differences were considered as statistically significant at *p* < 0.05.

## 4. Conclusions

In this study, the methanol extract from *A. indica* showed potential antioxidant activity and ovatodiolide purified from the extract displayed a dermatological effect against melanin production in B16F10 melanoma cells. This is the first report about the effect of *A. indica* methanol extract and ovatodiolide on melanin production. In the present study, it was found that *A. indica* methanol extract expresses antioxidant activity and ovatodiolide inhibits melanin synthesis significantly in a dose-dependent pattern. The results revealed that *A. indica* methanol extract and ovatodiolide could be added to skin care cosmetics through antioxidant activity and inhibitory action upon melanin production.
